# Implementation of a Digitally Enabled Care Pathway (Part 1): Impact on Clinical Outcomes and Associated Health Care Costs

**DOI:** 10.2196/13147

**Published:** 2019-07-15

**Authors:** Alistair Connell, Rosalind Raine, Peter Martin, Estela Capelas Barbosa, Stephen Morris, Claire Nightingale, Omid Sadeghi-Alavijeh, Dominic King, Alan Karthikesalingam, Cían Hughes, Trevor Back, Kareem Ayoub, Mustafa Suleyman, Gareth Jones, Jennifer Cross, Sarah Stanley, Mary Emerson, Charles Merrick, Geraint Rees, Hugh Montgomery, Christopher Laing

**Affiliations:** 1 Centre for Human Health and Performance University College London London United Kingdom; 2 DeepMind Health London United Kingdom; 3 Department of Applied Health Research University College London London United Kingdom; 4 Population Health Research Institute St George’s, University of London London United Kingdom; 5 Royal Free London NHS Foundation Trust London United Kingdom; 6 Faculty of Life Sciences University College London London United Kingdom

**Keywords:** nephrology, acute kidney injury

## Abstract

**Background:**

The development of acute kidney injury (AKI) in hospitalized patients is associated with adverse outcomes and increased health care costs. Simple *automated e-alerts* indicating its presence do not appear to improve outcomes, perhaps because of a lack of explicitly defined integration with a clinical response.

**Objective:**

We sought to test this hypothesis by evaluating the impact of a digitally enabled intervention on clinical outcomes and health care costs associated with AKI in hospitalized patients.

**Methods:**

We developed a care pathway comprising automated AKI detection, mobile clinician notification, in-app triage, and a protocolized specialist clinical response. We evaluated its impact by comparing data from pre- and postimplementation phases (May 2016 to January 2017 and May to September 2017, respectively) at the intervention site and another site not receiving the intervention. Clinical outcomes were analyzed using segmented regression analysis. The primary outcome was recovery of renal function to ≤120% of baseline by hospital discharge. Secondary clinical outcomes were mortality within 30 days of alert, progression of AKI stage, transfer to renal/intensive care units, hospital re-admission within 30 days of discharge, dependence on renal replacement therapy 30 days after discharge, and hospital-wide cardiac arrest rate. Time taken for specialist review of AKI alerts was measured. Impact on health care costs as defined by Patient-Level Information and Costing System data was evaluated using difference-in-differences (DID) analysis.

**Results:**

The median time to AKI alert review by a specialist was 14.0 min (interquartile range 1.0-60.0 min). There was no impact on the primary outcome (estimated odds ratio [OR] 1.00, 95% CI 0.58-1.71; *P*=.99). Although the hospital-wide cardiac arrest rate fell significantly at the intervention site (OR 0.55, 95% CI 0.38-0.76; *P*<.001), DID analysis with the comparator site was not significant (OR 1.13, 95% CI 0.63-1.99; *P*=.69). There was no impact on other secondary clinical outcomes. Mean health care costs per patient were reduced by £2123 (95% CI −£4024 to −£222; *P*=.03), not including costs of providing the technology.

**Conclusions:**

The digitally enabled clinical intervention to detect and treat AKI in hospitalized patients reduced health care costs and possibly reduced cardiac arrest rates. Its impact on other clinical outcomes and identification of the *active* components of the pathway requires clarification through evaluation across multiple sites.

## Introduction

### Background

Acute kidney injury (AKI)—a sudden decline in kidney function—can be caused by hypovolemia, infection (including severe sepsis), nephrotoxicity, primary renal diseases, and urinary tract obstruction [1]. Affecting more than 18% of hospitalized patients [2], it is associated with prolonged hospital stay, need for acute renal replacement therapy (RRT), or intensive care admission as well as the development of chronic kidney disease and the need for long-term dialysis [3-5]. Although AKI may be a marker of systemic physiological decompensation in acute illnesses (eg, sepsis, trauma, or high-risk surgery), AKI itself might directly cause additional deaths through, for instance, metabolic derangement or extracellular fluid volume overload [6]. Such impacts are expensive; AKI confers excess annual costs of £1 billion to the English National Health Service (NHS) [7]. Similar excess health care costs have been demonstrated in other health systems [8].

AKI management involves the identification and treatment of life-threatening complications and medical or surgical treatment of underlying cause, supportive care (including RRT, where necessary), and interventions to reduce risk of recurrence [9]. In response to poor outcomes and variations in care delivery [10], automated AKI alerts (using standardized definitions of its presence and severity based on increases in serum creatinine [11]) have been delivered using messages in electronic health record systems or through hospital pagers [12]. In England, this approach has been applied through the embedding of an AKI detection algorithm—The NHS Early Detection Algorithm, NHSEDA (Multimedia Appendix 1)—in laboratory information management systems [13]. However, evidence of the impact of electronic alerts in improving clinical outcomes in AKI is conflicting [14]. The greatest indications of improvement seem to occur when such detection systems are coupled with structured education and clinical intervention packages [15,16]. However, the delivery of such care pathways is challenging: AKI is common and of heterogeneous etiology; it presents in diverse settings; and it is normally, at least in its early stages, managed by a range of nonspecialist teams.

### Objectives

To address these issues, we developed a digitally enabled care pathway for AKI patients [17]. This uses a mobile app (Streams), which alerts a specialist response team to the presence of AKI in real time, simultaneously providing relevant clinical data in a user-friendly format and allowing communication of key triage decisions among team members. Members of the response team review patients using a care protocol that maps to best practice guidance [9].

We have reported the clinical impact of this digitally enabled care pathway on patients with AKI at the point of presentation to the emergency department (ED) [18]. The limited impacts we identified in this setting might reflect the difficulty of mitigating harm when AKI is well established or in the context of pathogeneses specific to community-acquired AKI. In this paper, we assess the impact of the care pathway on clinical outcomes for patients who develop AKI following hospital admission and on health care costs.

## Methods

### The Hospital Sites

The digital pathway was implemented at the Royal Free Hospital (RFH), a large (839 beds including a 34-bed intensive treatment unit [ITU]) hospital in north London, United Kingdom. It provides acute and emergency care as well as a range of specialist, regional inpatient services (eg, hepatology, HIV and infectious disease, amyloidosis, and vascular surgery) and has a large inpatient nephrology and renal transplant service.

For the purposes of our evaluation, we used a comparator site managed by the same health care provider organization (Royal Free London NHS Foundation Trust, RFLFT) in which the intervention was not implemented. Barnet General Hospital (BGH) is an acute general hospital with 459 beds. It has a 21-bed ITU that can provide acute RRT and a liaison nephrology service. Tertiary, specialist services are not provided on this site. A number of parallel improvement initiatives were ongoing at the comparator site during the study period, including a sepsis improvement project and an active deteriorating patients improvement program.

### Implementation

Blood tests, including serum creatinine, are routinely undertaken on hospitalized inpatients across all wards as directed by the treating clinicians. Historically, at both sites, blood tests would be reviewed in batches by the clinicians who ordered them. Results suggesting AKI would be telephoned to relevant wards by laboratory staff. Referral for nephrology assessment would be undertaken at the discretion of the clinical teams and using hospital pagers and phones. Cases would be prioritized and treated by the nephrology teams through assessment of referral information and results on desktop computers and through bedside review. The Patient at Risk and Resuscitation Team (PARRT) provides support to ward teams for patients deemed at risk of deterioration or who trigger existing, physiology-based early warning systems.

The digitally enabled AKI care pathway and the technical architecture of the Streams app have been described in detail previously [17], and the pre-existing and novel care pathways are shown in Multimedia Appendix 1. Members of the response team undertook training before implementation. Following implementation, Streams continuously applied the NHSEDA to creatinine results for all inpatients. Using iPhones (Apple Inc), the nephrology team was alerted to all potential cases of AKI, with their AKI stage and whether metabolic complications such as hyperkalemia were present. A curated dataset was provided, which included patients’ demographic characteristics, previously coded diagnoses, and relevant results. Filters excluded children, critical care and chronic dialysis patients, and those already under the care of the nephrology inpatient team from producing alerts. Cases were triaged in-app and, when clinical review was warranted, a best-practice care protocol was delivered (Multimedia Appendix 1). This was annotated and entered into the patient’s notes alongside an advisory sticker for key nursing actions (Multimedia Appendix 1). Recovery could be monitored in-app, and repeat AKI alerts were sent if AKI had not recovered after 48 hours or if AKI severity stage increased. Nephrology members received all AKI alerts, and PARRT members received alerts only for patients with stages 2 and 3 AKI. All team members could communicate in-app; triage responses and the outcome of clinical reviews were visible in the app to other team members. Implementation of the care pathway at the RFH used existing RFH PARRT and nephrology staff and did not result in expansion in staff numbers. A diagram outlining the pre- and postintervention care pathways is provided in Multimedia Appendix 1.

### Data Collection

At both sites, data from RFLFT hospital databases and those supporting Streams app relating to the intervention period (May to September 2017) were compared with those from a predeployment phase (May 2016 to January 2017). Data relating to patients in whom an AKI alert was generated on presentation to the hospital ED are reported elsewhere [18], and such patients were excluded from the analyses reported here.

Data collected and their sources are detailed in Table 1. The time frame to alert viewing was determined using data recorded by the Streams app. The presence of individual comorbidities and overall patient-specific Charlson comorbidity index score (which categorizes comorbidities based on the International Classification of Diseases diagnosis codes) were derived as per the method by Thygesen et al [19]. Patients were sorted into national quintiles of deprivation (quintile 1, least; quintile 5, most deprived) using Indices of Multiple Deprivation (IMD)—a measure combining 7 domains (income, employment, living environment, health, education skills and training deprivation and disability, barriers to housing and services, and crime) into a single deprivation score for a small area—by cross-referencing patients’ postcodes with the UK Government’s Indices of Deprivation 2015 dataset [20].

For the economic analysis, we used Payment Level Information and Costing System (PLICS) data supplied by the RFLFT. PLICS is a clinical costing system where costs are derived for each patient spell (ie, admission) by tracing resources used by an individual patient in diagnosis and treatment and calculating the expenditure on those resources using the actual costs incurred by the provider. PLICS has the advantage of including staffing costs and infrastructure absorbed costs. In our study, the PLICS data for hospitalized patients with AKI included the following components: total length of stay (including the length of stay in intensive care unit), pathology and radiology examinations, total theater time, theater cutting time, inpatient dialysis, and overhead costs. These data were analyzed at the spell level. We also obtained data on the costs associated with selected individual components of a spell, which we analyzed separately (ie, length of stay, pathology and radiology examinations, theater total time, and theater cutting time). However, individual cost components were based on tariffs and not fully absorbed costs. Furthermore, we could not obtain individual costs of inpatient dialysis. The final dataset used in the economic analysis comprised total and component-specific spell-level costs at the RFH and BGH, before and after the digitally enabled care pathway was introduced at the RFH.

### Evaluation of Impacts

**Figure 1:**
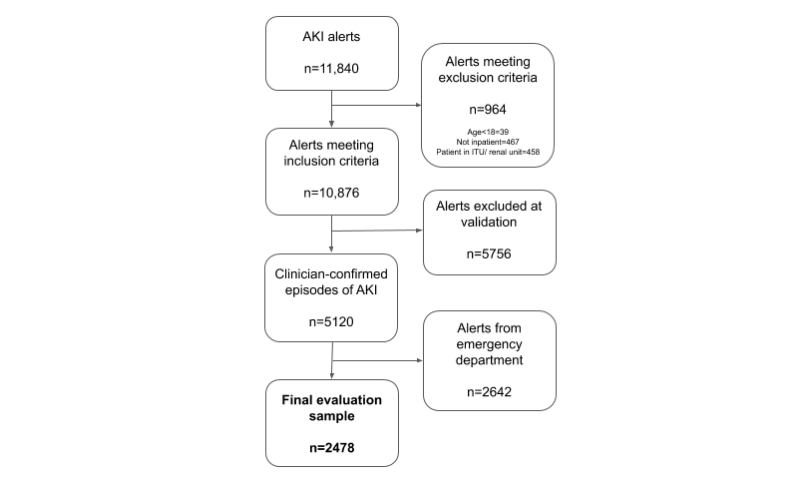
Defining the final evaluation sample. AKI: acute kidney injury; ITU: intensive treatment unit.

The primary outcome was recovery of renal function (return to a serum creatinine concentration within 120% of the baseline, as defined by the NHSEDA) before hospital discharge. Table 1 describes the predefined secondary endpoints. At both sites, NHSEDA was used to identify potential AKI cases. Because the NHSEDA can produce false positives [22], 2 authors (AC and CL) clinically validated all AKI alerts produced from all periods at both hospital sites. Only clinician-confirmed episodes of AKI were included in the analysis. In this paper, we report the outcomes of inpatients producing AKI alerts outside of the ED during the predeployment and deployment phases (Figure 1). The impact of the care pathway on cardiac arrests rate was measured on a hospital level, as it was not possible to ascertain which cardiac arrests occurred among patients with AKI.

**Table 1:** Definitions of each outcome and sources of data collected.

Data category and measure		Definition		Source of data	
**Sociodemographic characteristics**					
	Age		Age in years at the time of alert		HL7^a^ data aggregated within the Streams data processor
	Gender		Gender codes used in the NHS^b^ Data Dictionary [21]		HL7 data aggregated within the Streams data processor
	Ethnicity		Ethnicity category codes used in the NHS Data Dictionary [21]		HL7 data aggregated within the Streams data processor
	Comorbid disease		Presence of individual Charlson index comorbidities and overall Charlson score		HL7 data aggregated within the Streams data processor
	Deprivation		Index of Multiple Deprivation		Ministry of Housing, Communities and Local Government database
**Clinical outcomes**					
	Recovery of renal function		Return to <120% index creatinine (as defined by NHSEDA^c^) by the time of hospital discharge		HL7 data aggregated within the Streams data processor
	Time to recovery of renal function		The time from AKI^d^ alert to recovery of renal function (<120% index creatinine)		HL7 data aggregated within the Streams data processor
	Mortality		Death in 30 days following AKI alert		HL7 data aggregated within the Streams data processor
	Progression of AKI stage		Movement between AKI severity classes following AKI alert and before hospital discharge		HL7 data aggregated within the Streams data processor
	Admission to high acuity or specialist renal inpatient bed		Admission to acute kidney unit/high dependency unit/intensive treatment unit during index admission		HL7 data aggregated within the Streams data processor
	Requirement long-term renal replacement therapy		Use of hemofiltration/hemodiafiltration/hemodialysis/peritoneal dialysis in 30 days following hospital discharge date		RFH^e^ Nephrology Clinical Information Management System
	Length of stay		Time from AKI alert to hospital discharge		HL7 data aggregated within the Streams data processor
	Re-admission to hospital		Re-admission to hospital in 30 days following index admission discharge date		HL7 data aggregated within the Streams data processor
**Trust-wide metric**					
	Cardiac arrest rate		Number of cardiac arrests per 1000 bed days		Trust critical care nursing team logs
**Economic measures**					
	Costs per patient		Cost per patient per hospital spell		Payment Level Information and Costing System data and Payment by Results/local tariffs at the trust
**Process of care**					
	Time to alert review		Time from alert generation to alert viewing by a clinician		Data aggregated within the Streams data processor

^a^Health Level 7 (HL7) messages are used to transfer information between different health care information technology systems.

^b^NHS: National Health Service.

^c^NHSEDA: NHS Early Detection Algorithm.

^d^AKI: acute kidney injury.

^e^RFH: Royal Free Hospital.

### Statistical Analysis

All data were pseudonymized before transfer to the University College London (UCL) for analysis. Analyses were performed using R, version 3.4.3 (R core team) [23], and Stata MP version 14 (StataCorp) [24]. Segmented regression analysis estimated the intervention effect on the primary outcome (return to a serum creatinine concentration within 120% of the baseline, as defined by the NHSEDA) and 5 secondary outcome measures: mortality within 30 days of alert, progression of AKI stage, transfer to renal/intensive care units during admission, re-admission within 30 days of discharge, and dependence on RRT 30 days after discharge. Outcomes were measured as weekly proportions. We used binomial regression models with a logit link predicting the weekly rate of each outcome. Codes 1 and 0 were applied to the period after and before the intervention, respectively. The intervention and comparator sites were coded 1 and 0, respectively. The variable *time* denoted the week number (1 denoting the first week of the intervention period, and negative numbers denoting weeks in the preintervention period). The statistical model used was:

logit(y) = β_0_ + β_1_int + β_2_time + β_3_site + β_4_int x time + β_5_int x site + β_6_time x site β_7_int x time x site (1)

where the proportion of interest is denoted by *y*, the variables intervention, time, and site by *int*, *time,* and *site,* respectively (as defined previously), and the coefficients to be estimated by *β*_0_,...,. In addition, 2 coefficients evaluated the evidence for the intervention causing a step change in outcome: the effect of *intervention* estimates the step change in outcome at the start of the intervention period at the RFH. The interaction *site×intervention* estimates the difference-in-differences (DID) in the step change between the intervention and comparator hospital sites. We also evaluated evidence for a change in temporal trend in the outcome because of the intervention: the *time×intervention* interaction estimates the difference in temporal outcome trend between the intervention and preintervention periods at the RFL; the 3-way *time×site×intervention* interaction estimates the DID in the trend between the intervention and comparator sites.

For all models, we inspected the autocorrelation function (up to lag 15). No significant autocorrelation was detected in any model. At the point of protocol publication, it was not anticipated that we would be able to collect patient-level data relating to sociodemographic characteristics and comorbid disease.

To examine the robustness of our primary outcome analysis, we used binary logistic regression to perform a sensitivity analysis: the same model mentioned previously was used, except that (1) the outcome was defined at the patient level and (2) patient-level characteristics (age, sex, ethnicity category, index of multiple deprivation, AKI alert level, the presence of complications at the time of alert, and the presence of individual Charlson score comorbidities) were included as covariates to adjust for any differences in casemix between sites and within sites over time.

The Wilcoxon rank-sum test was used to analyze the time to creatinine recovery (where this occurred by hospital discharge). To allow for the effects of in-hospital death on this outcome, the effect of the intervention on the length of hospital stay was estimated by competing risk analysis [25]. To determine the effect of the intervention on the time to recognition of AKI, a survival analysis was performed. The Wilcoxon rank-sum and chi-square tests were used to analyze sociodemographic variables as appropriate.

A total of 500 alerts were selected randomly from all periods, and all sites were reviewed a second time to assess the reliability of case validation. Intra- and interrater reliability was determined using Cohen’s kappa coefficient (Multimedia Appendix 1).

The number of cardiac arrests was recorded monthly at both hospital sites. Data relating to those which occurred in the hospitals’ ED, cardiac catheterization laboratory, intensive care unit, coronary care unit or in patients who had a formal *not for resuscitation* order signed were not included in the monthly counts recorded at the hospital level. Poisson regression models with a log link and an offset variable adjusting for the number of admissions per month were used to estimate the intervention effect on this outcome. As data were collected monthly, there was a relative paucity of postintervention data points so that estimating the effect of the intervention on outcome trend was not possible. The statistical model was:

log(number of cardiac arrests) = β_0_ + β_1_int + β_2_site + β_3_int x site + log(number of admissions) (2)

Economic analyses used generalized linear models (GLMs) to estimate DID, where costs were defined at the spell level, and patient-level characteristics (age, sex, ethnicity category, IMD, the presence of complications at the time of alert, and the presence of individual Charlson score comorbidities, such as diabetes mellitus or congestive cardiac failure) were included as covariates so as to allow adjustment for any differences in casemix between sites and within sites over time. A GLM was specified using a gamma family and log link to account for data skewness. The model used was:

log(cost) = β_0_ + β_1_age + β_2_sex + β_3_ethnicity + β_4_imd + β_5_comp + β_6_CharlsonScore + β_7_time + β_8_site + β_9_time × site (3)

where *time* was defined in relation to the intervention. May to September 2016 was considered preintervention (t_1_), and May to September 2017 was considered postintervention (t_3_). For robustness checks, we also carried out a secondary analysis, where the preintervention period was May 2016 to January 2017. The coefficient *β*_9_ is the coefficient of interest, measuring the between-site DID, comparing the change over time at the RFH to the change over time at the BGH. We present predictive margins showing adjusted mean costs per spell at the RFH and BGH before and after the intervention was introduced at the RFH. We adjusted for clustering at the patient level to account for the possibility that patients may have had multiple spells.

### Ethical Approval

The digitally enabled care pathway constituted a new standard service at the RFH. The UCL Joint Research Office reviewed the study protocol and judged that the project fell under the remit of service evaluation as per guidance from the NHS Health Research Authority [26]. As such, no patient consent was required. The evaluation was registered with the RFH Audit Lead and Medical Director. An independent data monitoring committee (which included a patient member) reviewed all analyses before preparation for publication. A full list of committee members is provided in Multimedia Appendix 1.

## Results

Alerts produced for hospitalized patients during the intervention period were reviewed by a member of the specialist response team in a median time of 14.0 min (interquartile range [IQR] 1.0-60.0 min). At the intervention site, clinical validation of the 4392 and 2254 AKI alerts during predeployment (May 2016 to January 2017) and postdeployment (May to September 2017) phases, respectively, yielded 1760 and 919 inpatient AKI episodes in each phase. Of these, 56.5% (994/1960) and 52.2% (480/919), respectively, were located outside the ED. In the predeployment and postdeployment phases at the nonintervention site, clinical validation of the 2866 and 1364 alerts, respectively, yielded 1669 and 772 inpatient AKI episodes, with 39.2% (654/1669) and 45.3% (350/772) being located outside the ED.

Table 2 summarizes the sociodemographic and clinical characteristics of patients producing AKI alerts at both sites and periods. RFH inpatients were younger (median 72 vs 82 years, *P*<.001), less likely to be white (*P*<.001), and less deprived (*P*<.001) than at BGH. RFH patients had significantly less comorbidity (median [IQR] Charlson score 5.0 [3.0-8.0] vs 5.0 [4.0-8.0], *P*<.001). The proportion of patients with pre-existing renal disease was also lower (31.5% vs 37.2%, *P*<.001). Comparing the pre- and postintervention cohorts, there were some significant differences within the comparator site. At BGH, patients in the postintervention period had significantly more severe AKI (*P*=.01) and a higher burden of comorbid (*P*<.001) and renal disease (45.1% vs 32.9%, *P*<.001) than patients in the preintervention period.

**Table 2:** Sociodemographic and clinical characteristics of patients producing acute kidney injury alerts.

Variable		Hospital site/period				*P* value		
		RFH^a^		BGH^b^		RFH pre vs RFH post	BGH pre vs BGH post	All RFH vs all BGH
		Pre^c^	Post^d^	Pre	Post			
AKI^e^ alerts, n		994	480	654	350	—^f^	—	—
**Alert severity, n (%)**						.102	.01	.32
	AKI1	809 (81.4)	411 (85.6)	571 (87.3)	281 (80.3)			
	AKI2	127 (12.8)	44 (9.2)	60 (9.2)	47 (13.4)			
	AKI3	58 (5.8)	25 (5.2)	23 (3.5)	22 (6.3)			
Male, n (%)		541 (54.4)	257 (53.5)	331 (50.6)	186 (53.1)	.74	.48	.30
Age (years), median (interquartile range)		73.00 (58.00-84.00)	7.00 (57.00-83.00)	82.00 (73.00-88.00)	82.00 (73.25-88.75)	.14	.81	<.001
**Ethnicity, n (%)**						.09	.32	<.001
	White	625 (62.9)	281 (58.5)	512 (78.3)	274 (78.3)			
	Black or Black British	76 (7.7)	34 (7.1)	29 (4.4)	12 (3.4)			
	Asian or Asian British	110 (11.1)	52 (10.8)	60 (9.2)	25 (7.1)			
	Mixed	10 (1.0)	2 (0.42)	3 (0.5)	4 (1.1)			
	Other ethnic groups	173 (17.4)	111 (23.1)	50 (7.7)	35 (10.0)			

**Index of Multiple Deprivation, n (%)**						.87	.83	<.001
	Quintile 1 (least deprived)	184 (18.5)	84 (17.5)	42 (6.42)	25 (7.1)			
	Quintile 2	216 (21.7)	130 (27.1)	132 (20.2)	60 (17.1)			
	Quintile 3	233 (23.4)	89 (18.5)	183 (28.0)	111 (31.7)			
	Quintile 4	224 (22.5)	111 (23.1)	186 (28.4)	99 (28.3)			
	Quintile 5 (most deprived)	97 (9.8)	46 (9.6)	108 (16.5)	53 (15.1)			
	Unknown	40 (4.0)	20 (4.2)	3 (0.5)	2 (0.6)			
**Charlson Score, n (%)**						.49	<.001	<.001
	0	114 (11.5)	49 (10.2)	10 (1.5)	7 (2.0)			
	1	51 (5.13)	11 (2.3)	25 (3.8)	9 (2.6)			
	2	63 (6.3)	54 (11.2)	29 (4.4)	13 (3.7)			
	3	107 (1.8)	43 (9.0)	78 (11.9)	21 (6.0)			
	4	169 (17.0)	63 (13.1)	150 (22.9)	59 (16.9)			
	≥5	490 (49.3)	260 (54.2)	362 (55.4)	241 (68.9)			
Pre-existing renal disease present, n (%)		303 (30.5)	162 (33.8)	215 (32.9)	158 (45.1)	.23	<.001	<.001

^a^RFH: Royal Free Hospital.

^b^BGH: Barnet General Hospital.

^c^Pre: May 2016 to January 2017.

^d^Post: May 2017 to September 2017.

^e^AKI: acute kidney injury.

^f^Not applicable.

**Table 3:** Descriptive statistics of total cost per spell producing acute kidney injury alerts.

Statistics	Royal Free Hospital (£)		Barnet General Hospital (£)	
	Pre^a^	Post^b^	Pre	Post
Mean (SD)	12,015.24 (22,732.78)	10,154.92 (19,582.30)	7391.16 (14,346.27)	7108.88 (11,512.95)
Median	5640.50	4954.00	3712.50	3774.00
1st centile	166.00	207.00	160.00	199.00
25th centile	2391.50	2079.00	1424.00	1153.50
75th centile	13,208.50	10,567.00	8466.00	8897.00
99th centile	111,245.00	90,138.00	51,991.00	45,614.00

^a^Pre: May 2016 to January 2017.

^b^Post: May 2017 to September 2017.

Table 3 provides descriptive statistics of total costs per spell at each site before and after the intervention. Multimedia Appendix 1 shows the positively skewed distribution of these costs.

### Clinical Outcomes

Estimates from the models predicting clinical outcomes are reported in Tables 4-7, as far as they relate to the research hypotheses. All estimated model coefficients are reported in Multimedia Appendix 1.

**Table 4:** Results of segmented regression analyses for renal recovery and mortality.

Variable/interaction term	Renal recovery			Mortality		
	Beta	*P* value	OR^a^ (95% CI)	Beta	*P* value	OR (95% CI)
Intervention^b^	.00	.99	1.00 (0.58-1.71)	.17	.67	1.18 (0.55-2.52)
Site×intervention^c^	.22	.62	1.24 (0.53-2.92)	.06	.91	1.07 (0.36-3.15)
Time×intervention^d^	−.01	.61	0.99 (0.96-1.03)	.00	.89	1.00 (0.96-1.05)
Time×site×intervention^e^	−.03	.29	0.97 (0.92-1.03)	−.03	.44	0.97 (0.91-1.04)

^a^OR: odds ratio.

^b^The coefficient *intervention* provides an estimate of the difference in outcome between the intervention period and the preintervention period at RFH.

^c^The 2-way interaction *site×intervention* provides an estimate of the difference-in-difference between the 2 hospital sites.

^d^The 2-way interaction *time×intervention* provides an estimate of the difference in outcome trend over time in the intervention period compared with the preintervention period at RFH.

^e^The 3-way interaction *time×site×intervention* provides an estimate of the difference-in-difference in the trend between the sites.

**Table 5:** Results of segmented regression analyses for progression of acute kidney injury stage and admission to intensive treatment unit/renal unit.

Variable/interaction term	Progression of acute kidney injury stage			Admission to intensive treatment unit/renal unit			
	Beta	*P* value	OR^a^ (95% CI)		Beta	*P* value	OR (95% CI)
Intervention^b^	.67	.11	1.96 (0.86-4.47)		.40	.42	1.50 (0.57-4.00)
Site×intervention^c^	−.71	.27	0.49 (0.14-1.71)		−1.18	.18	0.31 (0.05-1.68)
Time×intervention^d^	−.01	.60	0.99 (0.93-1.04)		.02	.55	1.02 (0.96-1.08)
Time×site×intervention^e^	.04	.32	1.04 (0.96-1.13)		.07	.19	1.08 (0.97-1.20)

^a^OR: odds ratio.

^b^The coefficient *intervention* provides an estimate of the difference in outcome between the intervention period and the preintervention period at RFH.

^c^The 2-way interaction *site×intervention* provides an estimate of the difference-in-difference between the 2 hospital sites.

^d^The 2-way interaction *time×intervention* provides an estimate of the difference in outcome trend over time in the intervention period compared with the preintervention period at RFH.

^e^The 3-way interaction *time×site×intervention* provides an estimate of the difference-in-difference in the trend between the sites.

**Table 6:** Results of segmented regression analyses for hospital re-admission and renal replacement therapy use.

Variable/interaction term	Re-admission at 30 days			Renal replacement therapy use at 30 days		
	Beta	*P* value	OR^a^ (95% CI)	Beta	*P* value	OR (95% CI)
Intervention^b^	.20	.54	1.22 (0.65-2.29)	−3.32	.03	0.04 (0.00-0.62)
Site×intervention^c^	−.16	.77	0.86 (0.31-2.39)	−1.04	.99	0.35 (0-infinity)
Time×intervention^d^	−.03	.23	0.97 (0.93-1.02)	.00	.98	1.00 (0.83-1.23)
Time×site×intervention^e^	.01	.84	1.01 (0.94-1.08)	−17.62	.99	0.00 (0-infinity)

^a^OR: odds ratio.

^b^The coefficient *intervention* provides an estimate of the difference in outcome between the intervention period and the preintervention period at RFH.

^c^The 2-way interaction *site×intervention* provides an estimate of the difference-in-difference between the 2 hospital sites.

^d^The 2-way interaction *time×intervention* provides an estimate of the difference in outcome trend over time in the intervention period compared with the preintervention period at RFH.

^e^The 3-way interaction *time×site×intervention* provides an estimate of the difference-in-difference in the trend between the sites.

**Table 7:** Results of segmented regression analysis for hospital cardiac arrest rate

Variable/interaction term	Cardiac arrests		
	Beta	*P* value	OR (95% CI)
Intervention^a^	−.60	<.001	0.55 (0.38-0.76)
Site×intervention^b^	.12	.69	1.13 (0.63-1.99)

^a^The coefficient *intervention* provides an estimate of the difference in outcome between the intervention period and the preintervention period at RFH.

^b^The 2-way interaction *site×intervention* provides an estimate of the difference-in-difference between the 2 hospital sites.

#### Primary Outcome

We found no evidence for a step change in renal recovery rate (return to a serum creatinine concentration within 120% of the baseline) following the intervention at the RFH. The estimated odds ratio (OR) for the intervention step change was 1.00 (95% CI 0.58-1.71). There was also no evidence for a significant difference in step change of recovery rate between RFH and BGH (estimated OR 1.24, 95% CI 0.53-2.92; *P*=.62).

**Figure 2:**
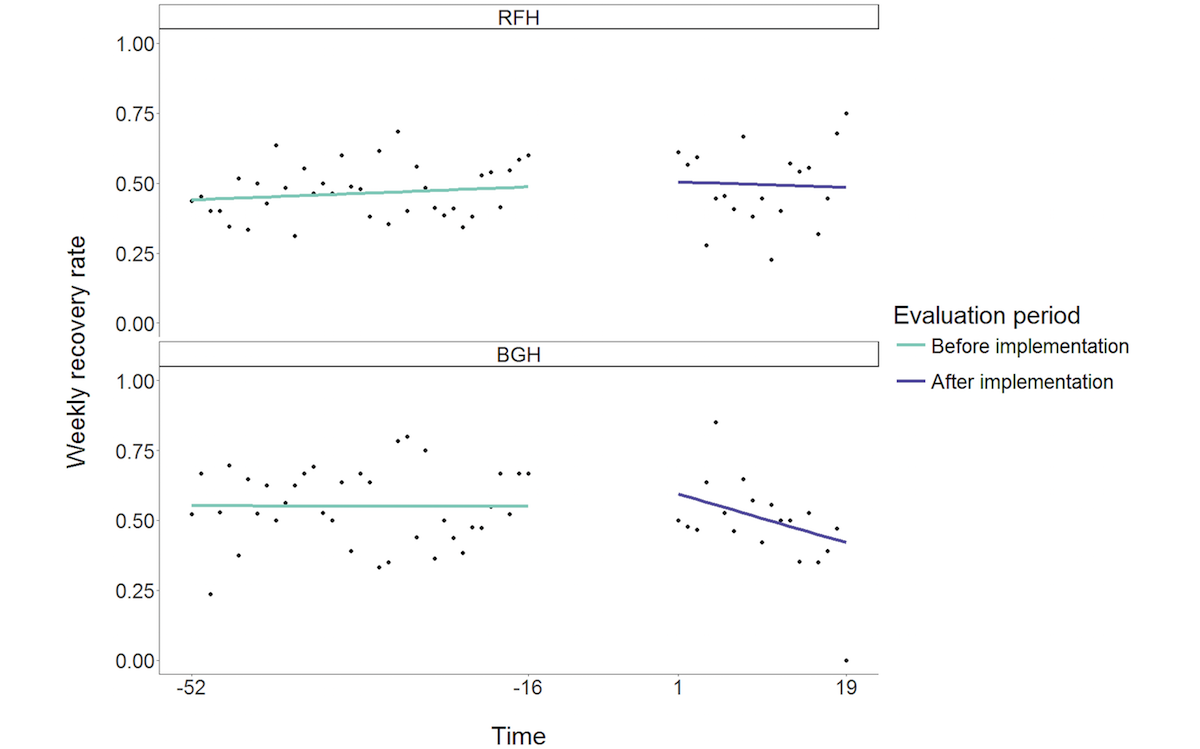
Weekly recovery rate at Royal Free Hospital (RFH) and Barnet General Hospital (BGH) before and after implementation of the care pathway. Individual data points reflect the rate of each outcome for a single week. Solid lines indicate fitted values from the modeling functions.

The model did not estimate a statistically significant change in the trend of renal recovery rates at RFH (estimated OR 0.99, 95% CI 0.96-1.03; *P*=.61), indicating that the trend in the intervention period at RFH was not significantly different to that in the preintervention period. There was also no significant difference in the trend change between sites (estimated OR 0.97, 95% CI 0.92-1.03; *P*=.29). The data and model predictions are illustrated in Figure 2. Model estimates from the sensitivity analysis controlling for differences in casemix did not differ substantially from the primary analysis model estimates (Multimedia Appendix 1), and none of the 4 examined estimated ORs were statistically significantly different from 1.

#### Secondary Clinical Outcomes

**Figure 3:**
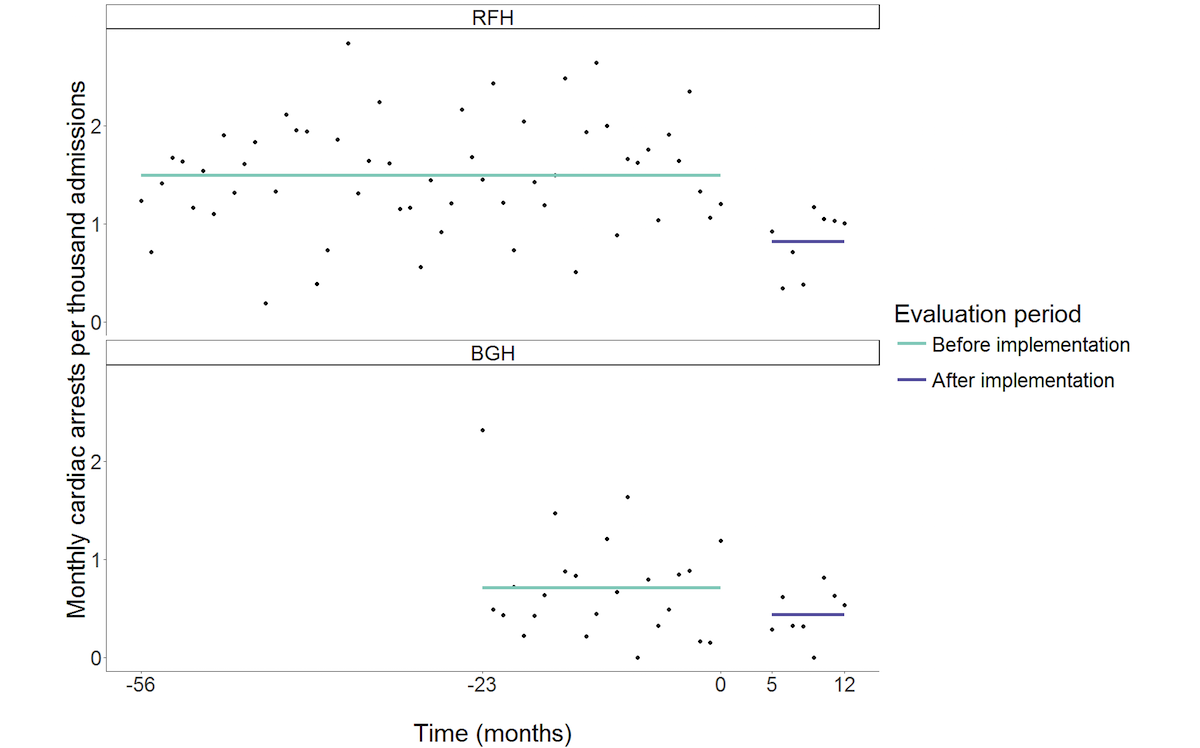
Cardiac arrests at Royal Free Hospital (RFH) and Barnet General Hospital (BGH). Individual data points reflect the rate of cardiac arrest per thousand admissions for a single month. Solid lines indicate fitted values from the modeling functions.

We found evidence for a reduction (step change) in the rate of cardiac arrest following the intervention at RFH (estimated OR 0.55, 95% CI 0.38-0.76; *P*<.001). However, we found no statistically significant difference in the step change between sites (OR 1.13, 95% CI 0.63-1.99; *P*=.69). The data and model predictions are shown in Figure 3.

We also found evidence for a reduction (step change) in the rates of RRT use at 30 days at the intervention site (estimated OR 0.04, 95% CI 0.00-0.62, *P*=.04). However, because RRT was a rare event, estimates for this outcome were not reliable (Tables 4-7 and Multimedia Appendix 1). For all other secondary outcomes, models did not provide statistically significant evidence for an impact of the intervention. The data and model predictions are shown in Multimedia Appendix 1.

We found no evidence for an effect of the intervention on time to renal recovery. At RFH, the median (IQR) time to renal recovery was 3.00 days (1.00-15.00 days) before and 4.00 days (1.00-12.00 days) after the introduction of the intervention (*P*=.61). At BGH, the median (IQR) time to renal recovery was 3.00 (1.00-13.00) and 3.00 (1.00-7.00) days, respectively (*P*=.100). Using competing risk analysis, a significant increase in length of stay was demonstrated at both RFH (*P*=.046) and BGH (*P*=.03) after implementation of the care pathway (Multimedia Appendix 1).

### Economic Outcomes

There was a significant reduction in adjusted mean costs per spell over time at the RFH but not at the BGH (Tables 8-10). There was a significant reduction in mean costs per spell at the RFH in the postimplementation period compared with the preintervention period over and above the (nonsignificant) change seen at the BGH: the DID was −£2123 per spell (95% CI=−£4024 to −£222, *P*=.03). For the specified secondary analysis including all periods, the DID was −£1631 per spell (95% CI=−£3218 to −£44, *P*=.04). No significant differences were noted in the analyses of the cost components (Multimedia Appendix 1).

**Table 8:** Results of economic analysis: Royal Free Hospital.

Time period	Preintervention (£)		Postintervention (£)		Difference (£)		*P* value
	Mean	95% CI	Mean	95% CI	Mean	95% CI	
Periods t1^a^ and t3^b^ only	12,176.52	10,996.53 to 13,356.50	9853.37	8840.91 to 10,865.82	−2323.15	−3843.90 to −802.41	.003
All periods	11,772.63	10,936.03 to 12,609.23	9761.59	8755.45 to 10,767.72	−2011.05	−3283.53 to −738.56	.002

^a^t1: May to September 2016.

^b^t3: May to September 2017.

**Table 9:** Results of economic analysis: Barnet General Hospital.

Time period	Preintervention (£)		Postintervention (£)		Difference (£)		*P* value
	Mean	95% CI	Mean	95% CI	Mean	95% CI	
Periods t1^a^ and t3^b^ only	7507.88	6589.77 to 8425.99	7307.27	6461.82 to 8152.71	−200.62	−1370.27 to 969.04	.74
All periods	7623.76	7007.67 to 8239.86	7243.58	6413.81 to 8073.35	−380.19	−1358.56 to 598.19	.45

^a^t1: May to September 2016.

^b^t3: May to September 2017.

**Table 10:** Results of economic analysis: difference-in-difference analysis of Royal Free Hospital and Barnet General Hospital.

Time period	Mean	95% CI	*P* value
Periods t1^a^ and t3^b^ only (£)	−2122.54	−4023.37 to −221.70	.03
All periods (£)	−1630.86	−3217.50 to −44.22	.04

^a^t1: May to September 2016.

^b^t3: May to September 2017.

## Discussion

### Principal Findings

The digitally enabled care pathway for the management of AKI in a large, acute hospital with a complex casemix resulted in no significant impact on the primary outcome of renal recovery or any of the other secondary clinical outcomes measured but was associated with a significant reduction in adjusted mean costs per patient admission. We did not include the costs of providing the technology, and therefore, it is not possible to judge whether or not it would be cost saving overall. Our results suggest that the digitally enabled care pathway would be cost saving, provided provision of the technology costs less than around £1600 per patient spell. The causes of the cost savings are unclear but are likely to be multifactorial, and further research to investigate these would be useful. The most important cost components contributing to this reduction (detailed in Multimedia Appendix 1) were length of stay and theater cutting time (which might itself be expected to play a role among patients requiring surgical intervention for AKI). There was a statistically significant reduction in the need for RRT at 30 days post-AKI; however, our model was not sufficiently reliable, given the low observed event rate of this outcome. The reduction in cardiac arrest rate needs to be viewed with caution because of the large number of hypothesis tests we conducted for our 6 prespecified secondary outcomes, and because this was a hospital-wide measure, this may have been influenced by other concurrently implemented initiatives. Furthermore, cardiac arrest rates also reduced at the comparator site. It is possible that both the RFH digital pathway and BGH’s quality improvement initiative were effective to some extent through different mechanisms.

There are several possible explanations for the lack of impact on renal recovery. First, this may reflect existing high standards of AKI care before implementation: 30-day mortality for preintervention patients at RFH was 14.9% compared with 18.1% nationally [27]. It is possible that our intervention may have delivered more benefit in hospitals with worse outcomes. Second, AKI arising during inpatient admission has been shown to have worse outcomes than that arising at emergency presentation [28]. This may be because AKI arising during hospital treatment may be harder to modify. Third, AKI detection using NHSEDA depends on an elevation of serum creatinine, the detection of which may lag many hours or even days after the time of renal insult [29]. In consequence, renal injury may be less modifiable by this stage, even using a rapid system of detection such as that described. Finally, it is possible that the Streams app may have had a greater impact were it to have been implemented as part of a different care pathway—perhaps, one that involved general physicians as well as specialty care.

An explanation for the possible effect of the intervention on rates of cardiac arrest emerged from qualitative data provided in our parallel paper [30]. Here, users suggested the care pathway not only enhanced early access to specialist care for deteriorating patients but also informed treatment escalation plans. The latter included institution of ceilings of care and *do not resuscitate* orders with patients and relatives. Both would be expected to contribute to a reduction in the recorded unexpected cardiac arrest rate.

### Comparison With Prior Work

Our data are consistent with recent reports of the benefits of e-alerting systems for AKI for patients and the wider health system. We have reported on the impact of the digitally enabled care pathway on processes of care and clinical outcomes for patients with AKI at the point of presentation to the ED. Implementation of the digitally enabled care pathway for these patients was associated with significant improvement in the reliability of AKI recognition, a reduction in time to recognize and adjust potentially nephrotoxic medications [18]. Our qualitative analysis [30] found that care pathway improved access to patient information and expedited early specialist care. Our results concur with other research findings: a recent study from Korea [31] suggested that e-alerting for inpatients improves AKI recognition and the number of patients receiving specialist review [31]. Moreover, 2 single-site quality improvement projects combining AKI alerts with care bundles and targeted staff education also improved recognition of AKI and the quality of inpatient care [32,33]. In addition, a large multicenter sequential period analysis of an alerting system warning clinicians of the possible presence of AKI next to the display of serum creatinine results resulted in a small but sustained decrease in in-hospital mortality, dialysis use, and length of stay [34]. However, similar to our research, it is unclear which components of these pathways influenced these outcomes. A number of mixed-methods analyses of e-alerting systems for AKI are still underway; results from the qualitative segments of the AKORDD [35] and TACKLING [36] studies are awaited.

### Strengths and Limitations

Our evaluation had a number of strengths. First, this is, to our knowledge, the first study to define the economic impact of implementing a digital innovation for AKI on health systems. Second, we clinically validated all NHSEDA AKI alerts before analysis and validated this process. Third, our inclusion of a comparator site follows best practice [37], ensuring transparency in the drawing of conclusions about the *active* components of our intervention.

Our evaluation also had several limitations. First, longer time frames and the inclusion of multiple intervention and comparator sites would have helped overcome the effect of differences in casemix in the pre- and postintervention period (identified in our single comparator site) and may have helped to clarify any added value of the integration of our digital innovation into the care pathway. This would also have allowed us to investigate the impact on specific patient subgroups and better understand if outcomes differed between different AKI stages. It is possible, for instance, that established severe AKI is far less responsive to intervention than the early disease. It is important that such issues are prospectively addressed in future studies. Longer time frames would also have allowed us to control for any seasonal changes in outcome, which are known to occur [38] and should be borne in mind in the design of future studies. It was not possible to collect cost data relating to the innovation of the intervention site, which should be included in any future cost-benefit analyses. Finally, although time to in-app AKI recognition and virtual review by a specialist was very rapid (median 14.0 min), comparable data from the preimplementation phase could not be collected as this process is integral to the Streams app.

### Conclusions

The digitally enabled AKI care pathway reduced inpatient health care costs and may also help reduce hospital-wide cardiac arrest rates: this result requires reanalysis in larger, multisite studies. Growing support for greater digitalization of health systems offers the opportunity to improve the quality and safety of care and to reduce its cost. However, prospective evaluation of the clinical and cost impacts of digital innovations within the context in which they are delivered will be key in delivering maximum utility for patients and health systems.

## Appendix

Multimedia Appendix 1 The National Health Service Early Detection Algorithm; acute kidney injury (AKI) care pathway; AKI care protocol; nursing advisory sticker; inter- and intra-operator variability analyses; Royal Free Hospital Data Monitoring Committee; distribution of costs per spell; results of segmented regression analyses; results from binary logistic regression analysis; graphs of secondary outcome data; cost component analyses.

## Abbreviations

AKI: acute kidney injury
BGH: Barnet General Hospital
DID: difference-in-differences
ED: emergency department
GLM: generalized linear model
IMD: Index of Multiple Deprivation
IQR: interquartile range
ITU: intensive treatment unit
NHS: National Health Service
NHSEDA: NHS Early Detection Algorithm
NIHR: National Institute for Health Research
OR: odds ratio
PARRT: Patient at Risk and Resuscitation Team
PLICS: Payment Level Information and Costing System
RFH: Royal Free Hospital
RFLFT: Royal Free London NHS Foundation Trust
RRT: renal replacement therapy
UCL: University College London
