# Supplementary material for: Implementation of a Digitally Enabled Care Pathway (Part 1): Impact on Clinical Outcomes and Associated Health Care Costs
Source: J Med Internet Res. 2019 Jul 15;21(7):e13147. doi: 10.2196/13147 (PMC6693300; doi:10.2196/13147)
Supplement: Multimedia Appendix 1 [file jmir_v21i7e13147_app1.pdf]

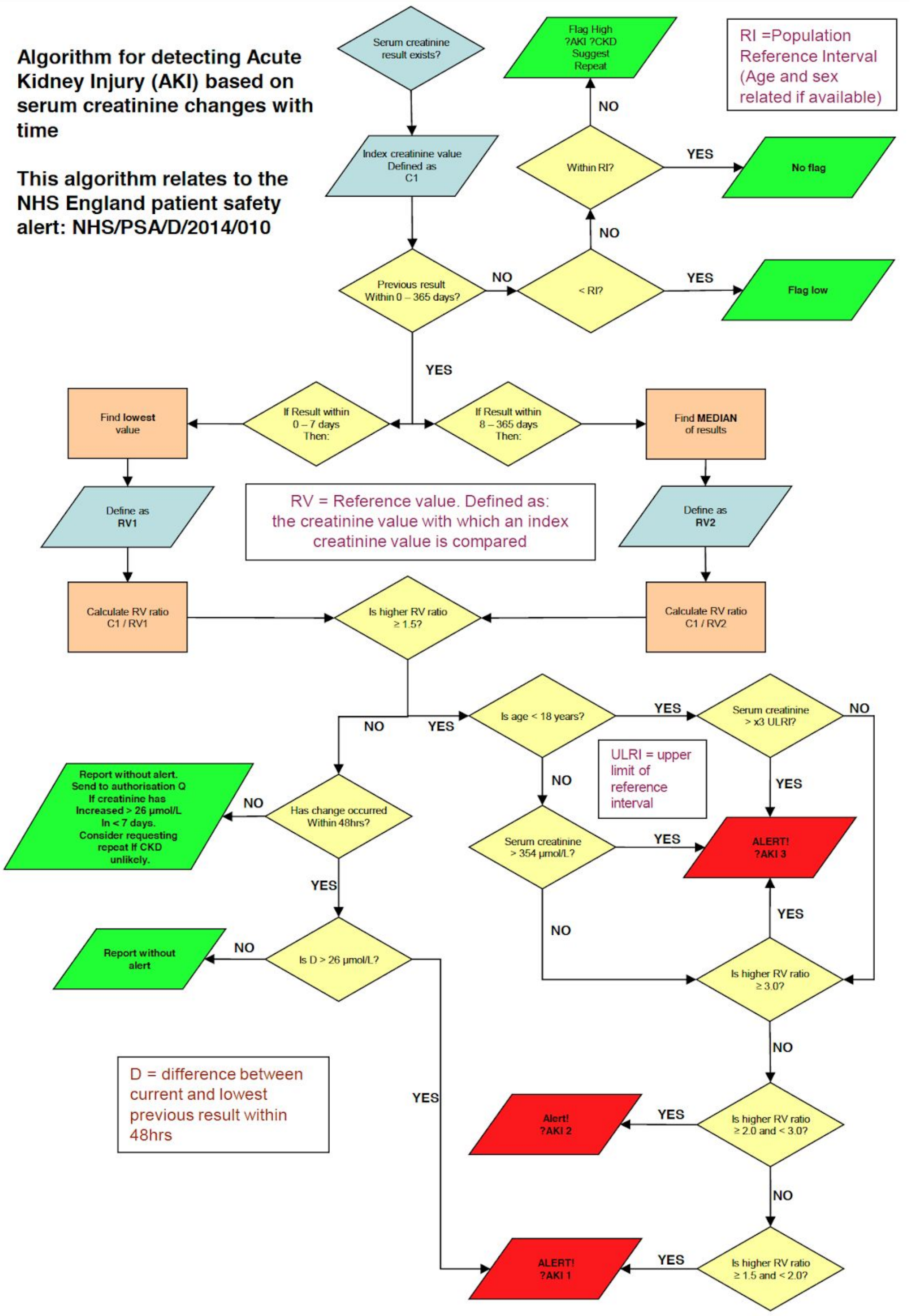

Figure 1: the NHS Early Detection Algorithm (NHSEDA)

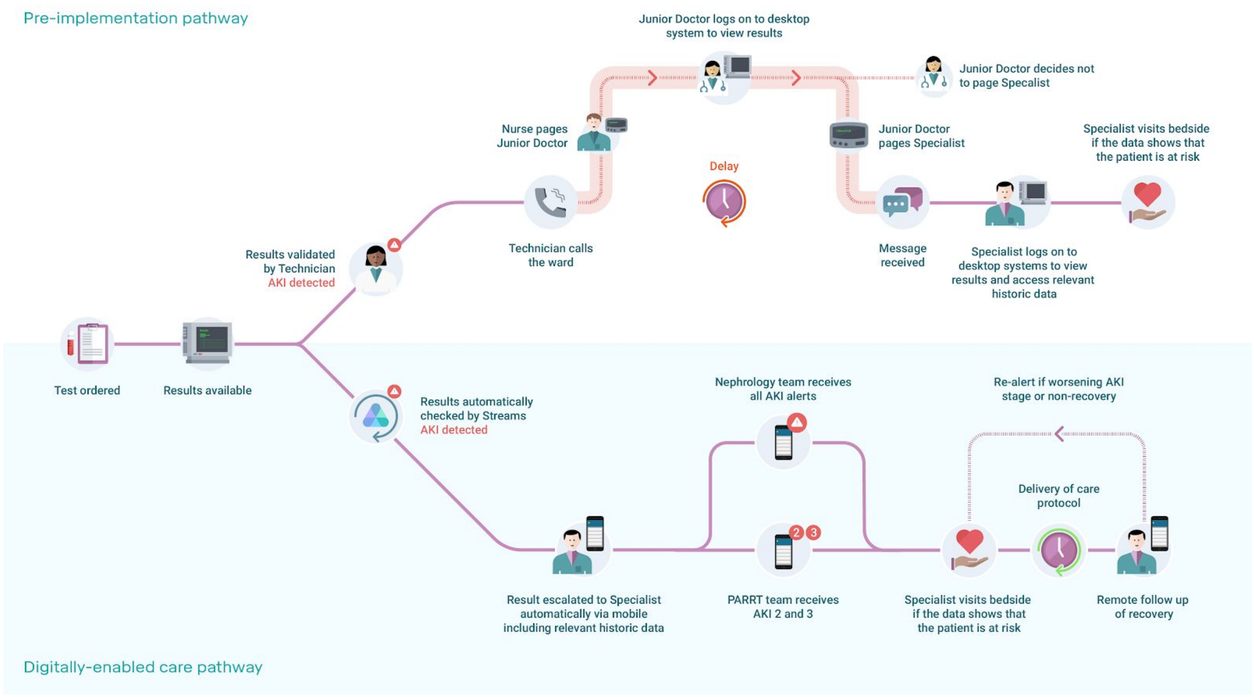

Figure 2: AKI care pathways before and after the introduction of the digitally-enabled care pathway

Royal Free London NHS
NHS Foundation Trust

This patient has generated an AKI alert on the Streams application.
The AKI response team are here to support and advise.
Ongoing management remains the responsibility of the home team unless otherwise agreed.

affix patient sticker here

We recommend the following actions to STOP-AKI:

**Management of life threatening complications of AKI**	**Critically ill:** Call PARRT (2525) or ITU (1030)	☐
	**Hyperkalaemia or acidosis:** commence medical therapy as per guidelines	☐
	**Fluid overload:** Commence diuretics, nitrates/oxygen (if necessary), fluid restriction	☐
**Sepsis and hypoperfusion**	**Sepsis:** complete Sepsis 6 care bundle	☐
	**Has an infection causing AKI:** send cultures, commence or escalate antibiotics	☐
	**Hypovolaemic:** Start bolus fluid protocol. Give 500mls crystalloid and reassess, repeat as necessary. Escalate to senior review after 2 litres bolus therapy	☐
	Commence maintenance IV fluids	☐
**Toxicity**	**Drug cessation or adjustment required**	☐
**Obstruction**	**Obstruction is possible and patient needs same day diagnostic renal USS** Please call Matteo Rossi for bedside USS on 07443101848. If out of hours then discuss with radiology (1462). If obstruction present please contact urology registrar on 1487 or on x39536	☐
**Primary Renal Disease**	**Perform urine dipstick**	☐
	If urine dip clear: order 'AKI diagnostic set (basic)' on Cerner	☐
	If blood or protein present: order 'AKI diagnostic set (glomerular)' on Cerner	☐

General advice

If in doubt, contact the AKI registrar on 07950860822 (day) or 07950843257 (night)

For guidelines and education, visit londonaki.net or download the London AKI app:

- Take 4 hourly observations & ensure an escalation plan is in place
- Commence a fluid balance chart, measure weights daily and set a daily fluid balance target
- Daily bloods: use 'AKI follow up' order set on Cerner and follow up to renal recovery
- Avoid contrast if possible. Consider prophylaxis where contrast absolutely necessary
- If renal function does not return to baseline at discharge, contact AKI registrar for advice

**Follow up**	We will only see if contacted by you or re-alerted in Streams due to worsening AKI	☐
	We will schedule a further review	☐
	We will take over care of patient	☐

TIME SEEN:______:______ DATE: ______/_____/_____ SIGNED:______________________________

Grade: Registrar ☐ Consultant ☐ NAME:______________________________

Figure 3: the care protocol

This patient has developed acute kidney injury (AKI)

You need to do the following to help your patient recover:

Do a urine dipstick

Start 4 hourly observations

Start a fluid balance chart
Ensure prescribed oral or intravenous fluid is administered.
Document inputs/ outputs/ 24 hour fluid balance and weight.

Escalate concerns
If concerned, get help. Call the medical team, and PARRT (2525).

Figure 4: nursing advisory sticker

	Operator 1	Operator 2
Operator 1	κ = 0.83 (0.76 - 0.90)	
Operator 2	κ = 0.75 (0.65 - 0.84)	κ = 0.79 (0.71 - 0.87)

Table 1: Inter- and intra-operator variability analyses

From the pool of alerts, a random selection of 250 from each operator were validated again by both. For each comparison pair, Cohen's kappa coefficient was calculated to establish inter- and intra-operator variability. 95% confidence intervals are shown in brackets

Chair

Prof. Kevin Moore
Professor of Hepatology, Royal Free Hospital

Patient representative

Michael Wise
Acute Kidney Injury National Programme Board

External members

Sir Nick Black
Professor of Health Services Research, London School of Hygiene and Tropical Medicine

Dr. Neil Ashman
Consultant Nephrologist and Deputy Managing Director, Barts Health

Royal Free Hospital Members

Dr. Jim Buckley
Consultant in Intensive Care Medicine

Dr. Nick Murch
Consultant in Acute Medicine

Dr. Jonathan Costello
Clinical Director, Emergency Medicine

Dr. Bimbi Fernando
Consultant in Transplant Surgery

Dr. Penny Smith
Consultant in Acute Medicine and Chief Medical Informatics Officer

Dr. Banwari Agarwal
Consultant in Intensive Care Medicine

Dr. Rupert Negus
Consultant in Acute Medicine and Gastroenterology

Figure 5: RFH Data Monitoring Committee

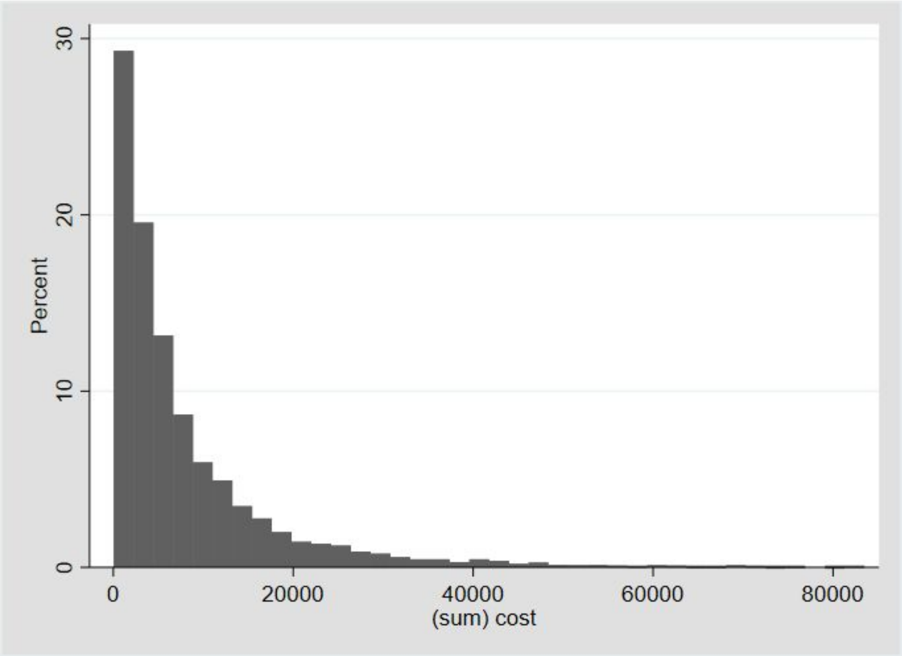

Figure 6: Distribution of cost per spell, across both sites and all times

	Renal recovery				Mortality			
	β	*P* value	OR	95% CI	β	*P* value	OR	95% CI
intervention	0.00	.99	1.00	(0.58-1.71)	0.17	.67	1.18	(0.55-2.52)
time	0.01	.39	1.01	(0.99-1.02)	0.00	.63	1.00	(0.98-1.01)
site	0.18	.58	1.20	(0.63-2.28)	0.73	.09	2.08	(0.90-4.79)
site×intervention	0.22	.62	1.24	(0.53-2.92)	0.06	.91	1.07	(0.36-3.15)
time×intervention	-0.01	.61	0.99	(0.96-1.03)	0.00	.89	1.00	(0.96-1.05)
time×site	-0.01	.58	0.99	(0.98-1.01)	0.01	.46	1.01	(0.99-1.03)
time×site×intervention	-0.03	.29	0.97	(0.92-1.03)	-0.03	.44	0.97	(0.91-1.04)

	Progression of AKI stage				Admission to ITU/Renal Unit			
	β	*P* value	OR	95% CI	β	*P* value	OR	95% CI
intervention	0.67	.11	1.96	(0.86-4.47)	0.40	.42	1.50	(0.57-4.00)
time	-0.01	.21	0.99	(0.97-1.01)	0.00	.86	1.00	(0.98-1.02)
site	0.52	.29	1.67	(0.64-4.38)	-0.17	.79	0.84	(0.24-2.85)
site×intervention	-0.71	.27	0.49	(0.14-1.71)	-1.18	.18	0.31	(0.05-1.68)
time×intervention	-0.01	.60	0.99	(0.93-1.04)	0.02	.55	1.02	(0.96-1.08)
time×site	0.01	.50	1.01	(0.98-1.04)	-0.01	.63	0.99	(0.96-1.03)
time×site×intervention	0.04	.32	1.04	(0.96-1.13)	0.07	.19	1.08	(0.97-1.20)

	Readmission at 30d				RRT use at 30d			
	β	*P* value	OR	95% CI	β	*P* value	OR	95% CI
intervention	0.20	.54	1.22	(0.65-2.29)	**-3.32**	**.03**	**0.04**	**(0.00-0.62)**
time	0.00	.91	1.00	(0.99-1.01)	**0.09**	**.03**	**1.09**	**(1.02-1.20)**
site	-0.13	.75	0.88	(0.40-1.91)	19.62	.33	3.33×10^{8}	$(0.04-8.27 \times 10^{30})$
site×intervention	-0.16	.77	0.86	(0.31-2.39)	-1.04	.99	0.35	$(1.61 \times 10^{-65}$-NA)
time×intervention	-0.03	.23	0.97	(0.93-1.02)	0.00	.98	1.00	(0.83-1.23)
time×site	0.00	.86	1.00	(0.98-1.02)	1.24	.33	3.44	(1.19-96.08)
time×site×intervention	0.01	.84	1.01	(0.94-1.08)	-17.62	.99	2.22×10^{-8}	(NA-4.86×10^{56})

	Cardiac arrests			
	**β**	***P* value**	**OR**	**95% CI**
intercept	-6.50			(-6.60 - -6.42)
intervention	**-0.60**	**<.001**	**0.55**	**(0.38-0.76)**
site	**-0.74**	**<.001**	**0.48**	**(0.38-0.59)**
site×intervention	0.12	.69	1.13	(0.63-1.99)

Table 2: Results of segmented regression analyses, including all estimated coefficients

The coefficient *intervention* provides an estimate of the difference in outcome between the intervention period and the pre-intervention period at RFH. The two-way interaction *site×intervention* provides an estimate of the difference-in-difference between the two hospital sites. The two-way interaction *time×intervention* provides an estimate of the difference in outcome trend over time in the intervention period compared to the pre-intervention period at RFH. The three-way interaction *time×site×intervention* provides an estimate of the difference-in-difference in the trend between the sites.

	Renal recovery			
	**β**	***P* value**	**OR**	**95% CI**
intervention	-0.10	.73	0.91	(0.52-1.58)
site×intervention	0.32	.47	1.38	(0.58-3.26)
time×intervention	-0.02	.40	0.98	(0.94-1.02)
time×site×intervention	-0.02	.42	0.98	0.92-1.03

Table 3: Results from binary logistic regression (sensitivity analysis)

The coefficient *intervention* provides an estimate of the difference in outcome between the intervention period and the pre-intervention period at RFH. The two-way interaction *site×intervention* provides an estimate of the difference-in-difference between the two hospital sites. The two-way interaction *time×intervention* provides an estimate of the difference in outcome trend over time in the intervention period compared to the pre-intervention period at RFH. The three-way interaction *time×site×intervention* provides an estimate of the difference-in-difference in the trend between the sites.

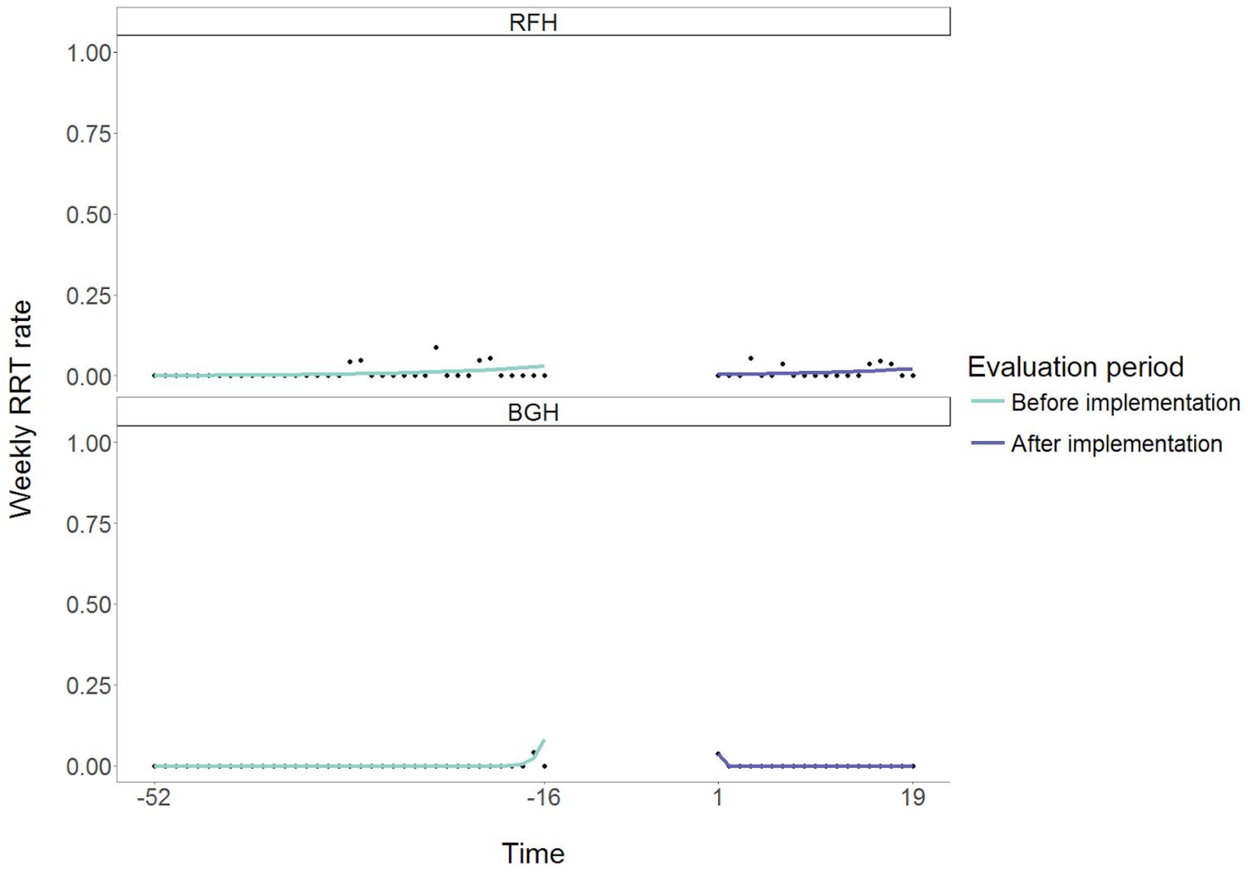

Figure 7: Weekly rates of 30-day dependence on renal replacement therapy at RFH and BGH before and after implementation of the care pathway

RFH = Royal Free Hospital, BGH = Barnet General Hospital.

Individual data points reflect the rate of each outcome for a single week.

Solid lines indicate fitted values from the modelling functions.

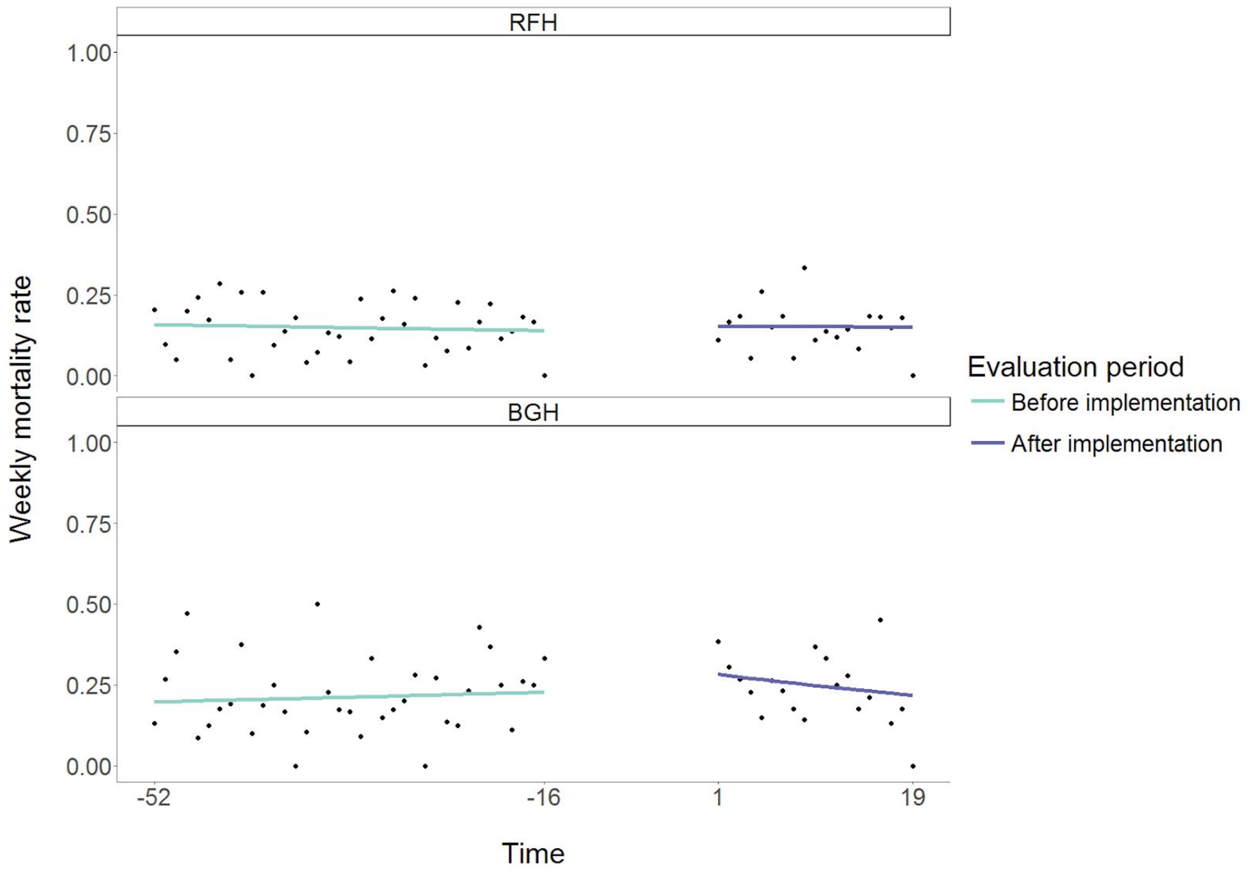

Figure 8: Weekly rates of mortality at RFH and BGH before and after implementation of the care pathway

RFH = Royal Free Hospital, BGH = Barnet General Hospital.

Individual data points reflect the rate of each outcome for a single week.

Solid lines indicate fitted values from the modelling functions.

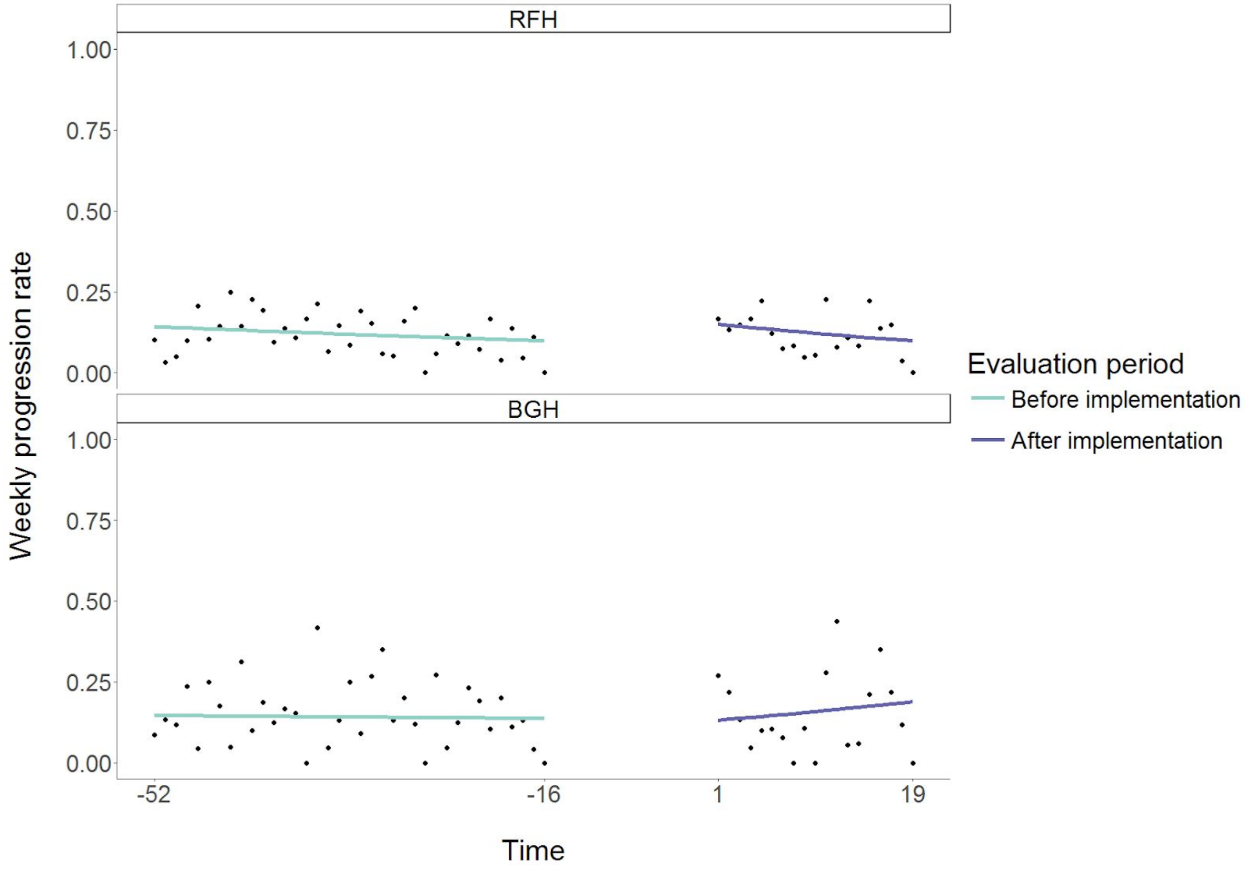

Figure 9: Weekly rates of AKI progression at RFH and BGH before and after implementation of the care pathway

RFH = Royal Free Hospital, BGH = Barnet General Hospital.

Individual data points reflect the rate of each outcome for a single week.

Solid lines indicate fitted values from the modelling functions.

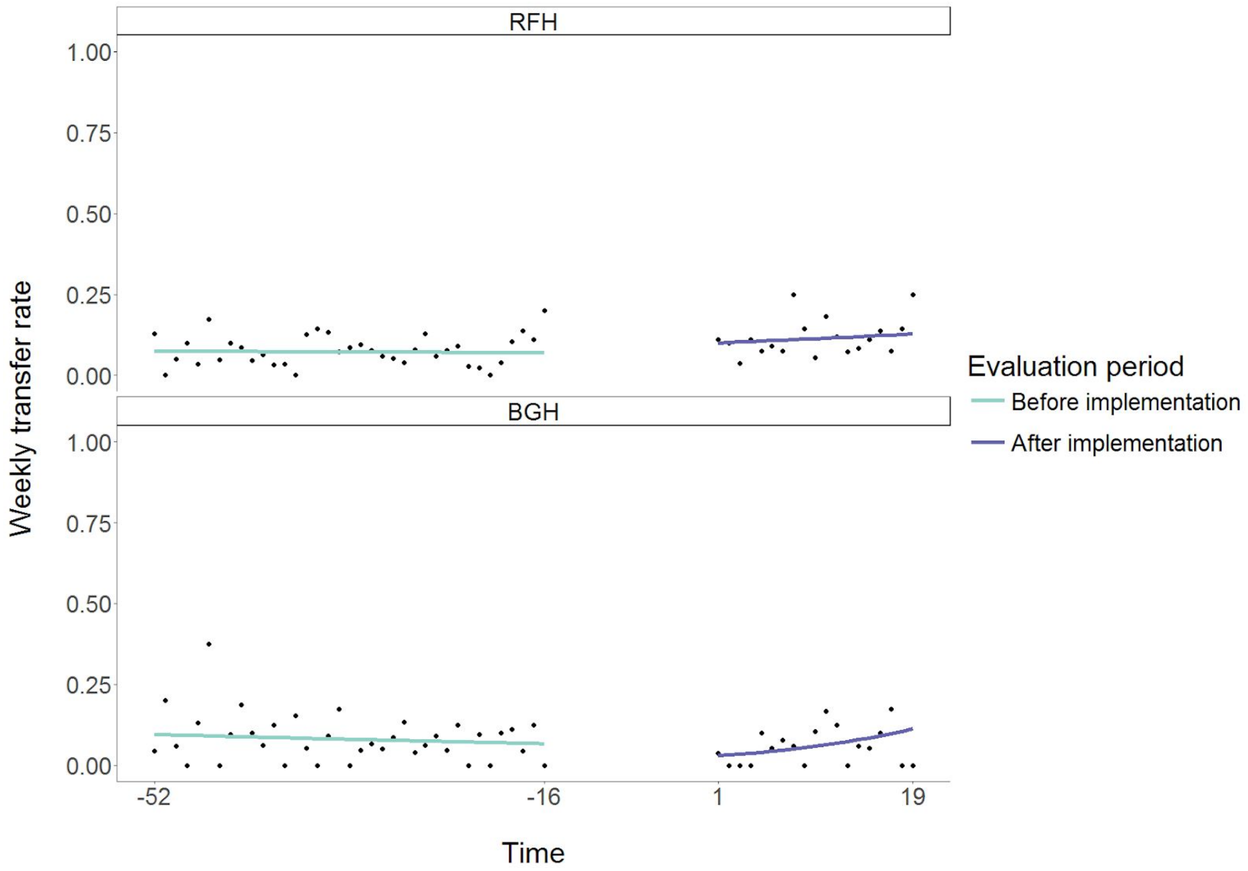

Figure 10: Weekly rates of transfer to ITU/ renal unit at RFH and BGH before and after implementation of the care pathway

RFH = Royal Free Hospital, BGH = Barnet General Hospital.

Individual data points reflect the rate of each outcome for a single week.

Solid lines indicate fitted values from the modelling functions.

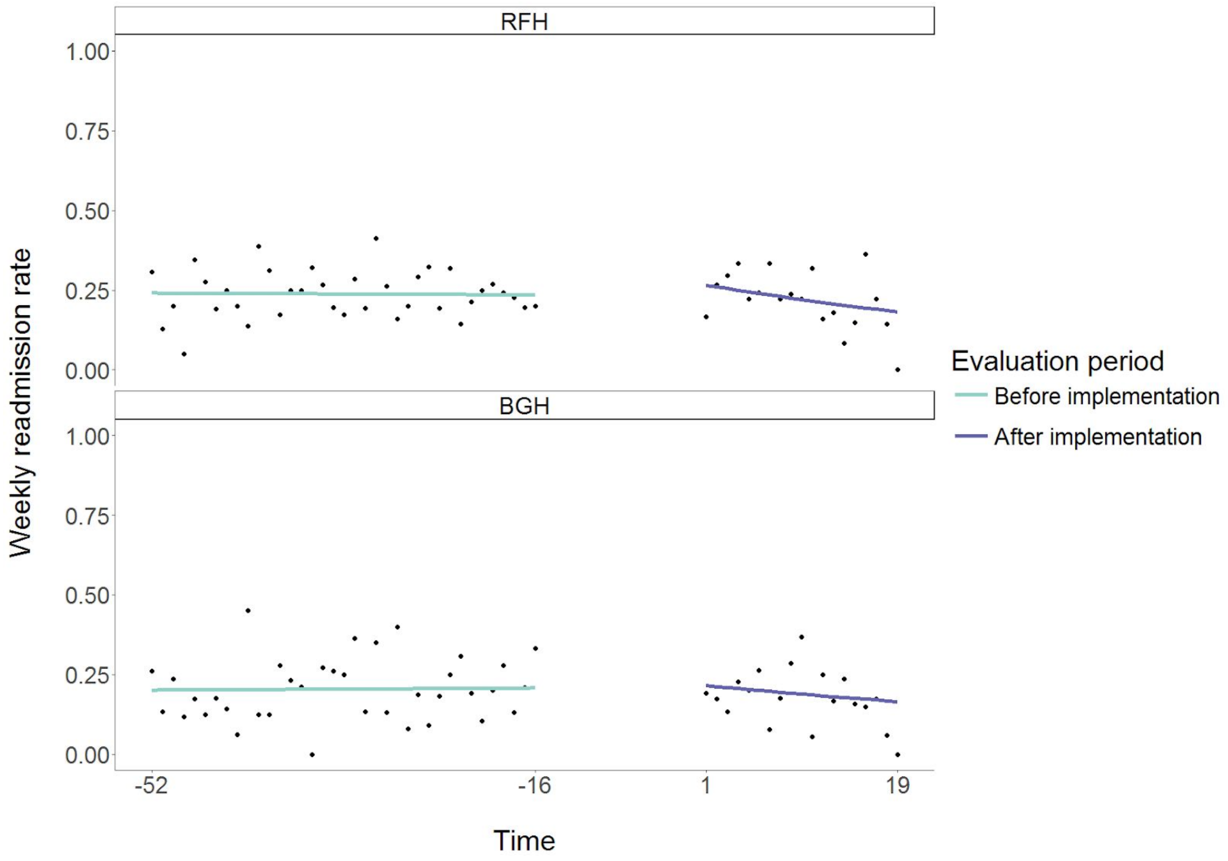

Figure 11: Weekly rates of readmission at RFH and BGH before and after implementation of the care pathway

RFH = Royal Free Hospital, BGH = Barnet General Hospital.

Individual data points reflect the rate of each outcome for a single week.

Solid lines indicate fitted values from the modelling functions.

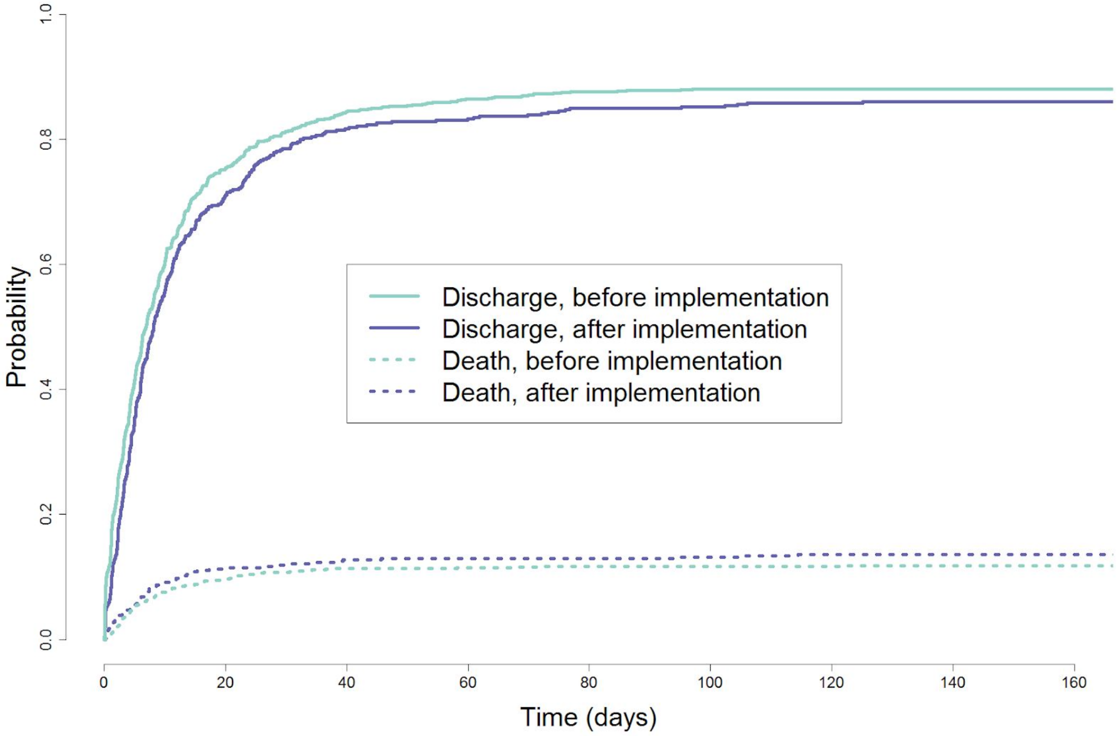

Figure 12: Plot of competing risk analysis for mortality and hospital discharge at RFH

Significant increase in LoS after implementation (*P*= .046). No significant difference in mortality after implementation (*P*=.32)

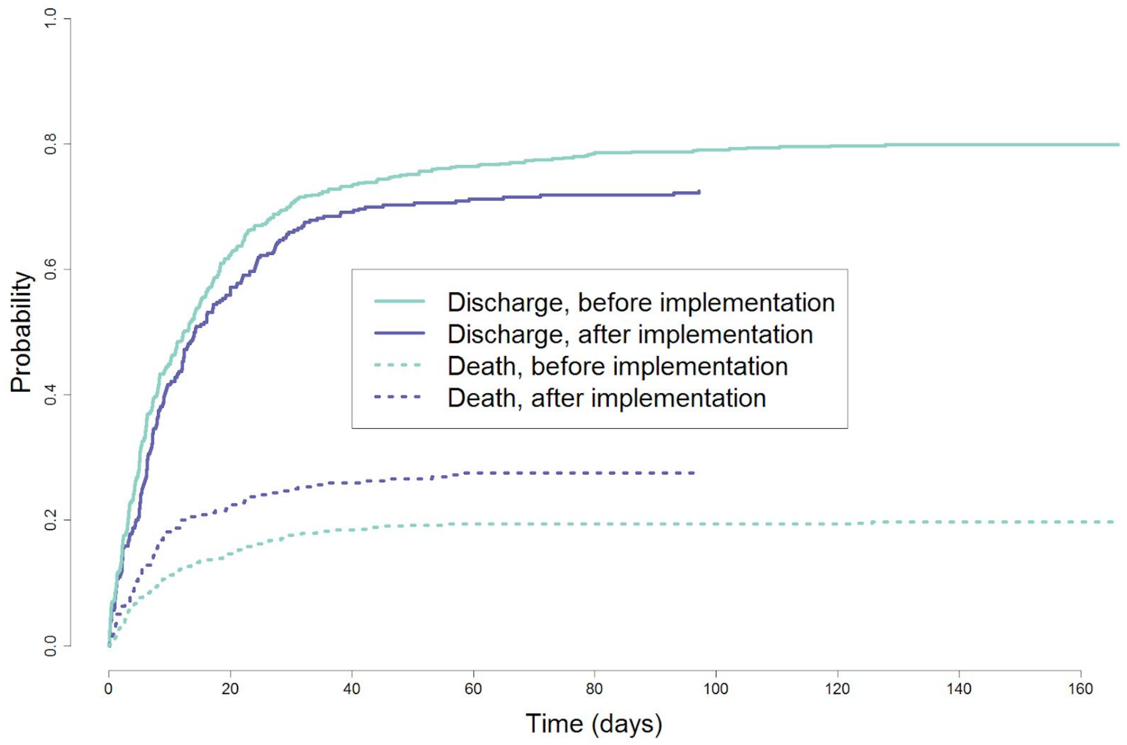

<u>Figure 13: Plot of competing risk analysis for mortality and hospital discharge at BGH</u>

Significant increase in LoS after implementation (*P*=.033). Significant increase in mortality after implementation (*P*=.003). NB: the model estimated odds ratio (OR) for the effects of the intervention on 30-day mortality was not significant (OR=2.08 (95%CI 0.90 - 4.79, *P*=.09).

RFH											
Component	Time period	Pre-intervention			Post-intervention			Difference			
		Mean	Lower CI	Upper CI	Mean	Lower CI	Upper CI	Mean	Lower CI	Upper CI	*P* value
Radiology exams	Periods t1&t3 only	£251.75	£228.98	£274.53	£219.32	£197.88	£240.76	**-£32.44**	**-£63.03**	**-£1.84**	**.04**
	All periods	£241.78	£225.89	£257.66	£215.37	£194.43	£236.30	**-£26.41**	**-£52.10**	**-£0.72**	**.04**
Pathology exams	Periods t1&t3 only	£534.45	£489.32	£579.58	£441.41	£402.10	£480.71	**-£93.04**	**-£151.76**	**-£34.32**	**.002**
	All periods	£507.40	£475.87	£538.93	£434.31	£395.92	£472.69	**-£73.09**	**-£121.53**	**-£24.65**	**.003**
Theatre cutting time	Periods t1&t3 only	£1,209.78	£982.57	£1,436.99	£949.20	£773.18	£1,125.23	**-£260.58**	**-£543.91**	**£22.75**	**.07**
	All periods	£1,106.97	£957.68	£1,256.26	£978.47	£792.31	£1,164.64	-£128.50	-£363.54	£106.54	.28
Theatre total time	Periods t1&t3 only	£901.27	£762.38	£1,040.16	£781.36	£651.20	£911.53	-£119.90	-£310.60	£70.79	.22
	All periods	£841.46	£745.99	£936.93	£798.94	£661.84	£936.04	-£42.52	-£209.88	£124.83	.62
Length of stay	Periods t1&t3 only	£6,412.47	£5,725.75	£7,099.20	£5,047.79	£4,490.96	£5,604.63	**-£1,364.68**	**-£2,227.27**	**-£502.10**	**.002**
	All periods	£6,312.34	£5,782.31	£6,842.37	£5,023.42	£4,464.65	£5,582.18	**-£1,288.92**	**-£2,018.84**	**-£559.01**	**.001**

BGH											
Component	Time period	Pre-intervention			Post-intervention			Difference			
		Mean	Lower CI	Upper CI	Mean	Lower CI	Upper CI	Mean	Lower CI	Upper CI	*P* value
Radiology exams	Periods t1&t3 only	£171.15	£154.52	£187.79	£157.83	£141.69	£173.96	-£13.33	-£35.80	£9.15	.25
	All periods	£172.87	£161.22	£184.52	£157.81	£141.77	£173.85	-£15.06	-£34.26	£4.13	.12
Pathology exams	Periods t1&t3 only	£618.41	£541.85	£694.97	£542.90	£483.49	£602.31	-£75.52	-£168.77	£17.74	.11
	All periods	£628.75	£579.91	£677.59	£536.59	£478.41	£594.78	**-£92.16**	**-£164.98**	**-£19.33**	**.01**
Theatre cutting time	Periods t1&t3 only	£717.52	£470.48	£964.55	£363.76	£239.74	£487.78	**-£353.75**	**-£615.93**	**-£91.57**	**.008**

	All periods	£570.91	£427.01	£714.81	£356.34	£229.38	£483.29	**-£214.57**	**-£401.39**	**-£27.76**	**.02**
Theatre total time	Periods t1&t3 only	£455.45	£330.19	£580.72	£292.41	£206.12	£378.71	**-£163.04**	**-£312.04**	**-£14.04**	**.03**
	All periods	£383.51	£305.42	£461.60	£292.34	£202.01	£382.66	-£91.18	-£211.39	£29.04	.14
Length of stay	Periods t1&t3 only	£5,469.91	£4,619.59	£6,320.22	£4,559.53	£3,991.79	£5,127.27	-£910.38	-£1,872.28	£51.53	.06
	All periods	£5,644.89	£5,099.14	£6,190.64	£4,511.97	£3,965.38	£5,058.56	**-£1,132.92**	**-£1,866.18**	**-£399.66**	**.002**

**Difference-in-difference**					
Component	Time period	Mean	Lower CI	Upper CI	*P* value
Radiology exams	Periods t1&t3 only	-£19.11	-£56.35	£18.12	.31
	All periods	-£11.35	-£42.97	£20.27	.48
Pathology exams	Periods t1&t3 only	-£17.53	-£127.13	£92.08	.75
	All periods	£19.07	-£67.80	£105.94	.67
Theatre cutting time	Periods t1&t3 only	£93.18	-£289.96	£476.31	.63
	All periods	£86.08	-£217.09	£389.25	.58
Theatre total time	Periods t1&t3 only	£43.13	-£196.92	£283.19	.72
	All periods	£48.65	-£158.95	£256.25	.65
Length of stay	Periods t1&t3 only	-£454.31	-£1,736.82	£828.21	.49
	All periods	-£156.00	-£1,170.46	£858.45	.76

Table 4: Cost components analyses

RFH = Royal Free Hospital, BGH = Barnet General Hospital. CI = Confidence Interval. t1 = May to September 2016; t2 = May to September 2017.
